# Caloric Restriction Alleviates CFA-Induced Inflammatory Pain *via* Elevating β-Hydroxybutyric Acid Expression and Restoring Autophagic Flux in the Spinal Cord

**DOI:** 10.3389/fnins.2022.828278

**Published:** 2022-04-28

**Authors:** Chang Liu, Xiaoting Zheng, Lifang Liu, Yun Hu, Qianyun Zhu, Jiawei Zhang, Huan Wang, Er-wei Gu, Zhilai Yang, Guanghong Xu

**Affiliations:** ^1^Department of Anesthesiology, First Affiliated Hospital, Anhui Medical University, Hefei, China; ^2^Department of Anesthesiology, Affiliated Chaohu Hospital, Anhui Medical University, Hefei, China; ^3^Key Laboratory of Anesthesia and Perioperative Medicine of Anhui Higher Education Institutes, Hefei, China

**Keywords:** caloric restriction, CFA, inflammatory pain, β-hydroxybutyric acid, autophagic flux

## Abstract

Inflammatory pain is the most common type of pain encountered in clinical practice; however, the currently available treatments are limited by insufficient efficacy and side effects. Therefore, new methods to relieve inflammatory pain targeting new mechanisms are urgently needed. Preclinical investigations have shown that CR (calorie restriction) exerts analgesic effects in neuropathic and cancer pain; however, the effect of CR on chronic inflammatory pain remains unknown. During calorie restriction, autophagy, a lysosome-dependent degradation process, can be activated to support cell survival. In the present study, we investigated the analgesic effects of CR on complete Freund’s adjuvant (CFA)-induced inflammatory pain. The accumulation of LC3-II and p62 showed impaired autophagic flux in the ipsilateral spinal cord of mice with CFA-induced inflammatory pain. CR alleviated mechanical allodynia and thermal hyperalgesia and reduced paw edema and pro-inflammatory factors following CFA administration. CR exerted an analgesic effect by restoring autophagic flux in the spinal cord. Regarding the mechanisms underlying the analgesic effects of CR, β-hydroxybutyric acid (BHB) was studied. CR increased BHB levels in the ipsilateral spinal cord. Furthermore, exogenous BHB administration exerted an analgesic effect by restoring autophagic flux in the spinal cords of CFA-induced inflammatory pain mice. Taken together, these results illustrated that CR relieved inflammatory pain by restoring autophagic flux in the spinal cord, while BHB controlled the benefits of CR, suggesting that CR or BHB might be a promising treatment for inflammatory pain.

## Introduction

As a rising health problem, chronic pain is expected to affect up to 30% of adults worldwide (Garland, 2014). Inflammatory pain is the most common type of chronic pain encountered in clinical practice (Barr et al., 2015). Chronic inflammatory pain is characterized by peripheral tissue damage and harmful stimuli that increase the response of the injured site and adjacent tissues, resulting in symptoms of hyperalgesia and allodynia (Descalzi et al., 2015). Some pro-inflammatory factors, such as interleukin-1β (IL-1β) and tumor necrosis factor-alpha (TNF-α), play an important role in the generation and maintenance of inflammatory pain, promoting central sensitization and hyperalgesia (Liu et al., 2021). Inflammatory pain not only seriously affects patients’ quality of life, but also creates a huge economic burden (Wyles et al., 2015). Currently available drugs for the treatment of inflammatory pain have various side effects, such as non-steroidal anti-inflammatory drugs (NSAIDs) and opioids. NSAIDs may cause gastrointestinal bleeding, chronic nephritis, and an increased risk of cardiovascular diseases (Lu et al., 2021). The analgesic properties of NSAIDs are insufficient in some patients, and their side effects limit their application in long-term therapy (Grosch et al., 2017). In addition, opioids may cause addiction. Therefore, new therapies to relieve inflammatory pain based on new mechanisms are urgently required.

Autophagy is a vital self-degradative cellular “cleanup” process that facilitates the removal of misfolded or aggregated proteins, as well as recycling of damaged cell components (Bagherniya et al., 2018). Autophagic flux is defined as the progression of autophagy, from the formation of autophagosomes to cargo delivery and lysosomal degradation by proteases. LC3 is cleaved from LC3I into a lower molecular weight LC3II and aggregates on to autophagosome membranes during the autophagy process. LC3II has been shown to be degraded for recycling during the last stages of autophagy, resulting in decreased levels. Sequestosome1 (SQSTM1/p62) is a protein substrate that is selectively incorporated into autophagosomes and degraded by autophagy. Blockade of autophagy flux is associated with increased p62 levels (Yoshii and Mizushima, 2017). Therefore, the adaptor protein p62 and LC3 are used to measure autophagic flux. Recent studies have shown that autophagy plays an important role in the occurrence and development of neuropathic pain (Liu et al., 2019; Hu et al., 2021; Li et al., 2021). Previous reports have demonstrated the dysfunction of autophagic flux in spinal nerve ligation (SNL), chronic constriction injury (CCI) and the spared nerve injury (SNI) models (Berliocchi et al., 2015). Autophagy interacts with inflammation and is believed to play an important role in inflammatory diseases (Matsuzawa-Ishimoto et al., 2018). Proinflammatory cytokines such as TNF-α and IL-1β are the earliest factors that cause inflammatory pain (Hwang et al., 2019). TNF-α and IL-1β interact with autophagy, which in turn regulates the expression of these proinflammatory cytokines, depending on the cellular context (Ge et al., 2018). Therefore, we investigated how autophagy regulated proinflammatory cytokines and pain behavior in chronic inflammatory pain. Our previous results showed that autophagic flux was impaired in the spinal cord of rats with inflammatory pain. Therefore, restoring autophagic flux may be an important strategy to improve chronic inflammatory pain.

The use of autophagy agonists to systemically enhance autophagy can improve some diseases, but can also cause a wide range of side effects (Yang and Zhang, 2020). Therefore, new strategies with higher efficacy and safety are urgently required. Calorie restriction (CR) activates autophagy and has less impact on animal health. Calorie restriction refers to a 10–30% reduction in food intake compared with *ad libitum* intake in the absence of malnutrition (Lee and Min, 2013). Several studies have shown that CR exerts neuroprotective effects. For example, CR has a significant benefit for prevalent neurodegenerative disorders, such as Alzheimer’s disease, Huntington’s disease, and Parkinson’s disease (Martin et al., 2006). In addition, CR has been reported to be effective in moderating the expression of some inflammatory markers that are upregulated during aging (Ugochukwu and Figgers, 2007). IL-1β and TNF-α are pro-inflammatory mediators known to be released by microglia in the CFA model, therefore, as a molecular correlate of CR efficacy, we tested whether CR was able to reduce it. Additionally, many studies have shown that CR is associated with analgesia. In a formalin-induced acute inflammatory pain model, mice with CR showed reduced pain response (Hargraves and Hentall, 2005), and recent studies have shown that CR can improve neuropathic pain (Liu et al., 2018; De Angelis et al., 2020). However, no studies have yet shown whether CR could improve CFA-induced chronic inflammatory pain. Therefore, we investigated the effects of CR on pain perception and autophagy in mice with CFA-induced chronic inflammatory pain. We used CQ (a lysosomal inhibitor) to block autophagic flux to determine whether CR improves chronic inflammatory pain by restoring autophagic flux. Our data indicated that CR improved CFA-induced inflammatory pain and restored autophagic flux in the spinal cord, and that CQ antagonized the analgesic effect of CR.

The ketone body β-hydroxybutyrate (BHB) is synthesized from fatty acids in the liver and serve as alternative energy sources when the supply of glucose is not enough for the body’s energetic need (Newman and Verdin, 2017). BHB levels can be markedly elevated under abnormal conditions such as caloric restriction, fasting, or a low-carbohydrate ketogenic diet, etc. (Huang et al., 2018). A recent study revealed that BHB served as a metabolic intermediary of CR and controlled the benefits of CR for improving ischemia and reperfusion triggered liver injury (Miyauchi et al., 2019). Our data indicated that CR elevated BHB levels in the spinal cord, and BHB might control the benefits of CR in CFA-induced inflammatory pain. In brief, we want to illustrate whether CR can alleviates CFA-induced inflammatory pain by regulating autophagy in the spinal cord and the role of BHB in CR therapy. Our results show that calorie restriction or BHB may be an effective and feasible method to improve chronic inflammatory pain.

## Materials and Methods

### Animals

Male C57BL/6 mice (6–8 weeks old) were purchased from Shanghai SLAC Laboratory Co., Ltd. All animals were housed under a 12 h light and dark cycle (temperature, 22–24^°^C), with free access to water and food. Mice were allowed to adapt to the conditions for at least 7 days before all experiments. All experimental procedures and animal welfare experiments were carried out in accordance with the Ethical Regulation on the Care and Use of Laboratory Animals of Anhui Medical University, and were approved by the school committee for animal experiments.

### Dietary Regimen

**FIGURE 1:**
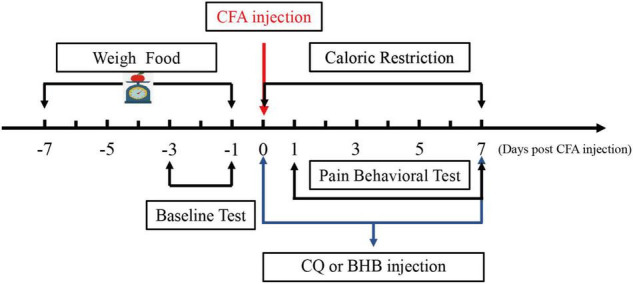
Schematic diagram showing the time points of CR administration and the CQ, BHB injections.

A standard regimen was used for CR. All mice received the same commercial laboratory pellets (Xietong Feed Co., Jiangsu, China). Mice in the control and CFA groups were provided feed *ad libitum* (AL). We measured the daily food intake of mice (approximately 3.2 g per day) for 1 week before CFA injection, and mice in the CR and CFA + CR groups received 70% of the average food intake following CFA injection until the mice were sacrificed. Food was weighed and provided to the animals in the CR and CFA + CR groups daily, approximately 1 h before the start of the dark cycle, to avoid disrupting the circadian rhythm (Liu et al., 2018). During this period, body weight was regularly measured (Figure 1).

### Inflammatory Pain Model

To construct the inflammatory pain model, mice received an intraplantar injection of CFA (20 μl) in their left hind paw. Saline was used as the control instead of CFA.

### Drug Treatment

Chloroquine (CQ) was obtained from Sigma-Aldrich (St. Louis, MO, United States) and dissolved in saline for intraperitoneal (i.p.) administration. CQ solution was injected intraperitoneally 1 h before CFA injection at a dose of 50 mg/kg/day, and subsequently administered every day at the same time until the mice were sacrificed (Zhang et al., 2017; Weng et al., 2019). Equivalent dose of normal saline was administered to the CFA + CR group.

DL-BHB (Sigma-Aldrich) was dissolved in PBS and adjusted to a pH of 7.5. BHB was injected intraperitoneally (i.p.) 1 h before CFA administration at a dose of 300 mg/kg/d (3% w/v, 10 ml/kg) and then administered every day at the same time until the mice were sacrificed. The doses of BHB were chosen based on previous studies (Yamanashi et al., 2017; Hazem et al., 2018).

### Paw Edema

Paw edema induced by the CFA injection was considered as the paw thickness. We used a digital caliper to measure the maximal dorsal-ventral paw thickness (*n* = 10–12). Paw thickness was recorded immediately before CFA injection (baseline) and then on days 1, 3, 5, and 7 after CFA injection. The caliper was consistently placed in the center of the left hind paw (Justino et al., 2020).

### Behavioral Tests

Behavioral tests were performed between 11:00 and 15:00 to minimize any possible influence of the satiety effect of recent feeding and the potential reward of feeding soon after the behavioral test (Liu et al., 2018). Before the behavioral test, the animals were habituated to the testing conditions for 3 days. Mechanical withdrawal threshold (MWT) and thermal withdrawal latency (TWL) tests were performed before CFA injection and on days 1, 3, 5, and 7 following injection (*n* = 9–12). Before each test, the mice were acclimatized to the surroundings for 30 min. The behavioral investigators were blinded to the drug administration conditions.

In the MWT test, each mouse was placed in a small plexiglass cage (10 × 15 × 15 cm) with a metal mesh floor. Mechanical allodynia was assessed using an electronic von Frey device (2091 series; IITC Life Science Inc., United States). A positive response was defined as flinching or withdrawal of the left hind paw and the force that elicited the withdrawal reflex was recorded. The tests were repeated three times with a 5 min interval between tests, and the mean force was used.

To quantitatively assess TWL, mice were placed on the glass surface of a thermal testing apparatus (Model 336, IITC/Life Science Instruments, Woodland Hills, CA, United States). The movable heat stimulator was moved to focus the heat on the central plantar surface of the left hind paw through the glass plate. Nociceptive endpoints were defined by observation of characteristic lifting or licking of the hind paw, and the time to the endpoint was considered as the paw TWL. Each test session included three thermal stimuli at 5 min intervals, and the mean latency was used. A cutoff time of 20 s was set to avoid tissue damage.

### Specimen Preparation

The mice were sacrificed after the behavioral tests. The ipsilateral spinal cord segments (L3–5) were removed and immediately stored at −80^°^C for subsequent experiments (Xie et al., 2021).

### Western Blot

The mice (*n* = 4) were sacrificed on the seventh day after CFA injection. The ipsilateral spinal cord was mixed with RIPA, and homogenized, then the homogenate was centrifuged at 10,000 rpm at 4^°^C for 10 min to obtain the supernatant. Proteins extracted from the ipsilateral spinal cord were subjected to 13.5% sodium dodecyl sulfate (SDS) polyacrylamide gel electrophoresis and transferred to a polyvinylidene fluoride membrane. Thereafter, the membrane was incubated in blocking buffer [5% skim milk in Tris-buffered saline with polyoxyethylene sorbitan monolaurate (TBS-T)] for 1 h at room temperature. The membranes were incubated with the following primary antibodies at 4^°^C overnight: P62 (1:1,000; Abcam), LC3 (1:1,000; Abcam), and Anti- β-actin (1:1,000; Abcam). After washing, membranes were incubated with secondary antibodies (1:10,000; Bio-Rad) for 1 h at 37^°^C. The membranes were incubated with ECL reagents and visualized using a chemiluminescence instrument (Amersham Imager 600).

### Enzyme-Linked Immunosorbent Assay

Mice (*n* = 4) were sacrificed on the third day after CFA injection, and the days for enzyme-linked immunosorbent assay (ELISA) were chosen based on previous studies (Zucoloto et al., 2019). The ipsilateral spinal cord was mixed with ice-cold PBS, homogenized, and the homogenate was centrifuged at 10,000 rpm at 4^°^C for 10 min to obtain the supernatant. The levels of IL-1β and TNF-α were detected using ELISA kits from Cusabio (Wuhan, China), according to the manufacturer’s instructions.

### Measurement of Spinal β-Hydroxybutyrate Concentration

Mice (*n* = 4) were sacrificed on the seventh day after CFA administration. Ipsilateral spinal cord samples were rinsed with phosphate-buffered saline (PBS) to remove any red blood cells or clots. Spinal samples were homogenized in beta-hydroxybutyrate assay buffer (Item No. MAK041A; Sigma, United States) and centrifuged at 13,000 × g to obtain the supernatant. The BHB concentration in the supernatant was assayed using a BHB assay kit (Item NoMAK041, Sigma) according to the manufacturer’s protocol.

### Immunofluorescence Staining

The mice (*n* = 3) were deeply anesthetized with a lethal dose of sodium pentobarbital (70 mg/kg body weight, i.p.) and perfused with 20 ml PBS, followed by 20 ml of 4% paraformaldehyde on the seventh day after CFA injection. The L3-5 spinal cords segments were removed and post fixed in 4% paraformaldehyde at 4^°^C overnight. The samples were immersed in 30% phosphate-buffered sucrose for 24 h, and then blocked in Tissue-Tekr OCT compound at −80^°^C. Immunofluorescence was performed on 8 μm thick L3–5 transverse spinal sections. Sections were collected on microscopic slides, air-dried, and processed for immunofluorescence staining. The spinal cord sections were washed three times for 5 min with PBS, permeabilized in 0.1% Triton X-100 for 10 min, washed three times for 5 min with PBS, and blocked for 1 h with 2% fetal bovine serum (FBS). Subsequently, the spinal cord sections were incubated with primary antibodies at 4^°^C overnight, LC3 antibody (1:100 Abcam), and then incubated with a AlexaFluor568-conjugated secondary antibodies (1:200, Abcam) for 1 h at 37^°^C in the dark. Finally, spinal cord sections were observed under a fluorescence microscope (Olympus BX53, Olympus, Japan).

### Statistical Analysis

All data are expressed as mean ± *SD*. Statistical analysis was performed using the GraphPad Prism software (version 7.0). Data regarding pain behaviors were analyzed using two-way repeated measures analysis of variance (treatment time), followed by Bonferroni *post-hoc* testing. Data from western blotting, ELISA, and BHB concentration were analyzed using one-way analysis of variance, followed by Tukey’s *post-hoc* test. *P < 0.05, ^**^P < 0.01, and ^***^P < 0.001 were considered statistically significant.

## Results

### Calorie Restriction Improves CFA-Induced Inflammatory Pain

**FIGURE 2:**
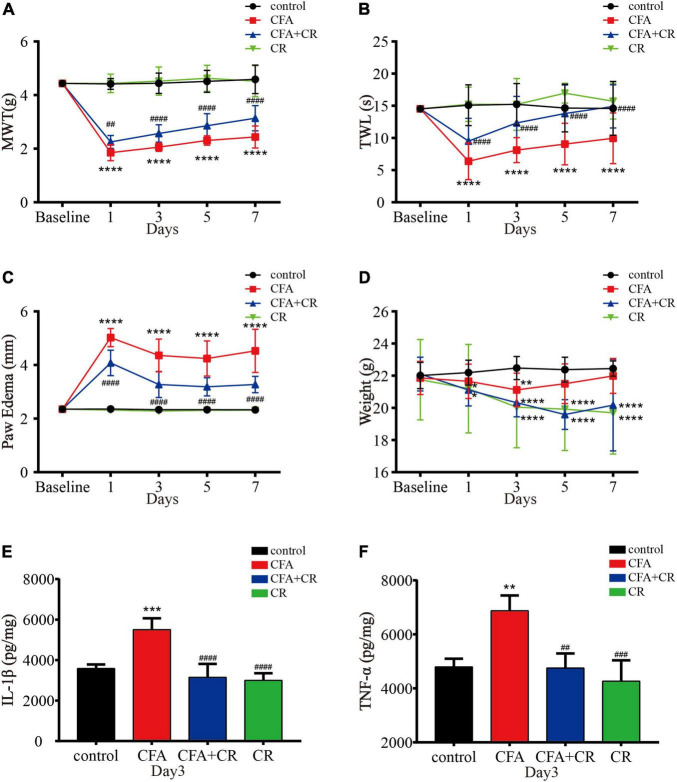
CR attenuates CFA-induced inflammatory pain. **(A)** Results of the mechanical withdrawal threshold (MWT) tests anssd **(B)** thermal withdrawal latency (TWL) tests of mice in the control, CFA, CFA + CR, and CR groups. **(C)** The paw edema and **(D)** the Weigh tests of mice in the four groups. Values are expressed as the mean ± *SD* and were analyzed by two-way repeated measures analysis of variance, followed by Bonferroni *post-hoc* testing, *n* = 12 per group. ^****^P < 0.0001, ^**^P < 0.01, *P < 0.05, compared to control group; ^####^*P* < 0.0001, ^##^*P* < 0.01, compared to the CFA group. **(E)** The level of IL-1β and **(F)** the level of TNF-α were measured in the four groups. Values are expressed as the mean ± *SD* and were analyzed by one-way analysis of variance, followed by a Turkey’s *post-hoc* test, *n* = 4 per group. ^***^P < 0.001, ^**^P < 0.01, compared to control group; ^####^*P* < 0.0001, ^###^*P* < 0.001, ^##^*P* < 0.01, compared to the CFA group.

MWT and TWL were measured to evaluate the effect of CR on mechanical allodynia and thermal hyperalgesia after CFA injection. As shown in Figures 2A,B, CR attenuated CFA-induced mechanical allodynia and thermal hyperalgesia, whereas only CR had no influence on mechanical allodynia and thermal hyperalgesia in control mice. Paw edema was measured to evaluate the anti-inflammatory activity of CR (Almeida et al., 2020). As shown in Figure 2C, CFA induced significant left paw edema. Paw edema reached a peak on the first day after CFA administration, and then began to recede gradually in a time-dependent manner. In the CFA + CR group, CR attenuated CFA-induced paw edema. However, CR alone did not change the hind paw thickness in control mice without CFA administration. The levels of proinflammatory cytokines were also measured to study the anti-inflammatory activity of CR. As indicated in Figures 2E,F, the concentrations of the pro-inflammatory cytokines IL-1β (Figure 2E) and TNF-α (Figure 2F) increased in the ipsilateral spinal cord 3 days after CFA injection. In brief, CR administration counteracted the CFA-induced elevation of inflammatory cytokine levels.

### Calorie Restriction Ameliorates Autophagic Flux in the Spinal Cord After CFA Administration

**FIGURE 3:**
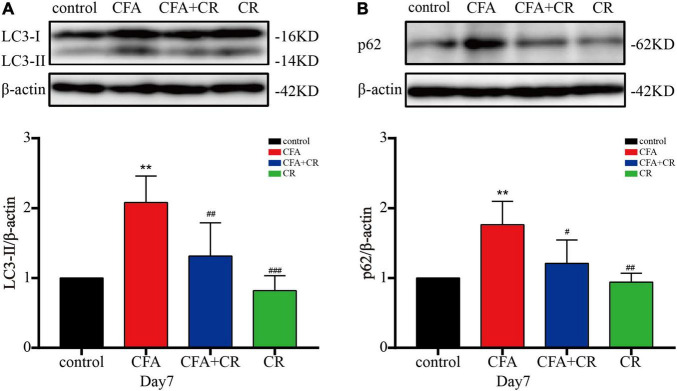
CR ameliorates autophagic flux in the spinal cord after CFA injection. Protein expressions of LC3-II **(A)** and p62 **(B)** of mice in the control, CFA, CFA + CR and CR groups, respectively. Values are expressed as mean ± *SD* and were analyzed by one-way analysis of variance, followed by a Turkey’s *post-hoc* test, *n* = 4 per group. ^**^P < 0.01, compared to control group; ^###^*P* < 0.001, ^##^*P* < 0.01, ^#^*P* < 0.05, compared to the CFA group.

To further test the integrity of autophagic flux, sequestosome1 (SQSTM1/p62) and LC3-II were evaluated by western blotting. The results showed that CFA treatment significantly increased p62 and LC3-II levels. Compared with mice in the CFA group, the expression of p62 and LC3-II was decreased in the CFA + CR group (Figures 3A,B). In addition, immunofluorescence was used to detect the expression of LC3 in the ipsilateral spinal cord. Compared to the control and CR groups, more LC3 positive cells were observed in the L3–5 spinal cord of mice in the CFA group. However, CR decreased the number of LC3 positive cells after CFA administration (CFA group vs. CFA + CR group) (Supplementary Figures 2A,B). These results suggest the blockage of autophagic flux in the ipsilateral spinal cord of mice with CFA-induced inflammatory pain, whereas CR may restore impaired autophagic flux.

### Calorie Restriction Improves CFA-Induced Inflammatory Pain by Restoring Autophagic Flux

**FIGURE 4:**
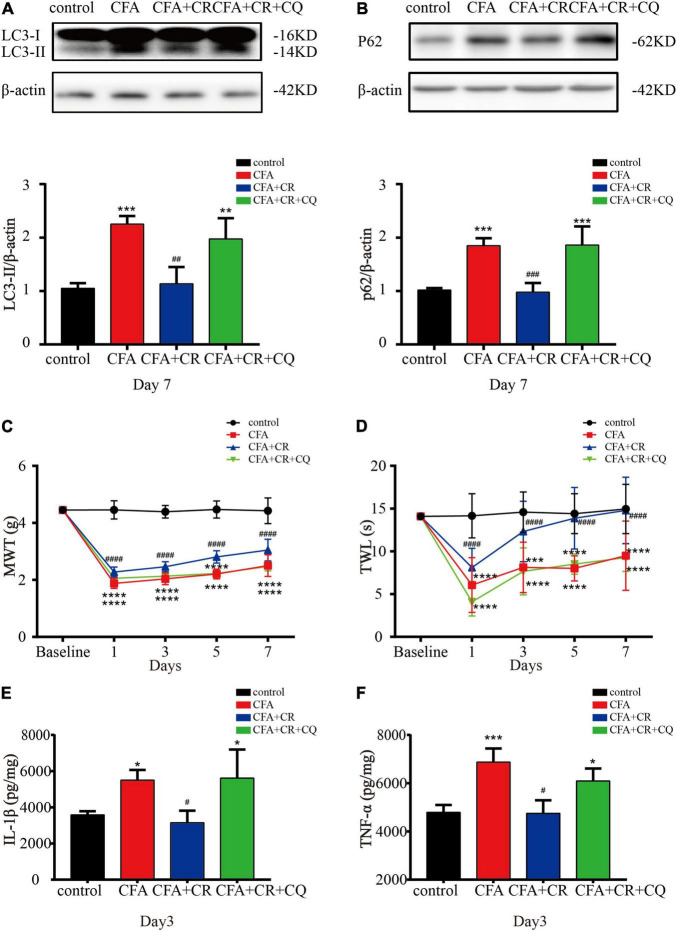
CR improves CFA-induced inflammatory pain by restoring autophagic flux. Protein expressions of LC3-II **(A)** and p62 **(B)** of mice in the control group, CFA group, CFA + CR group and CFA + CR + CQ group. Values are expressed as the mean ± *SD* and were analyzed by one-way analysis of variance, followed by a Turkey’s *post-hoc* test, *n* = 4 per group. ^***^P < 0.001, ^**^P < 0.01, compared to control group;^###^*P* < 0.001, ^##^*P* < 0.01, compared to the CFA + CR + CQ group. **(C)** The mechanical withdrawal threshold (MWT) tests and **(D)** thermal withdrawal latency (TWL) tests of mice in the four groups. Values are expressed as the mean ± *SD* and were analyzed by two-way repeated measures analysis of variance followed by Bonferroni *post-hoc* testing, *n* = 10 per group. ^****^P < 0.0001, ^***^P < 0.001, compared to control group; ^####^*P* < 0.0001, compared to the CFA + CR + CQ group. The level of IL-1β **(E)** and TNF-α **(F)** in the four groups. Values are expressed as the mean ± *SD* and were analyzed by one-way analysis of variance, followed by a Turkey’s *post-hoc* test, *n* = 4 per group. ^***^P < 0.001, *P < 0.05, compared to control group;^#^*P* < 0.05, compared to the CFA + CR + CQ group.

To determine the exact role of autophagic flux in CFA-induced inflammatory pain, and to determine if CR functions by restoring autophagic flux, CQ, a lysosomal inhibitor, was used to block autophagic flux. The results showed that CQ co-administration abrogated the decrease in LC3-II and p62 levels induced by CR (Figures 4A,B), confirming the blockage of autophagic flux by CQ. Furthermore, by applying the MWT and TWL tests, we found that the MWT and TWL were reduced in the CFA + CR + CQ group compared with those in the CFA + CR group (Figures 4C,D), indicating that the analgesic effect of CR therapy on pain perception depended on restoring autophagic flux. Moreover, we measured the levels of pro-inflammatory factors in the spinal cord. The results showed that the levels of IL-1β and TNF-α were significantly higher in the CFA + CR + CQ group than in the CFA + CR group (Figures 4E,F). These results demonstrated that CR could improve CFA-induced inflammatory pain by promoting autophagic flux.

### β-Hydroxybutyric Acid Controls the Benefits of Calorie Restriction

**FIGURE 5:**
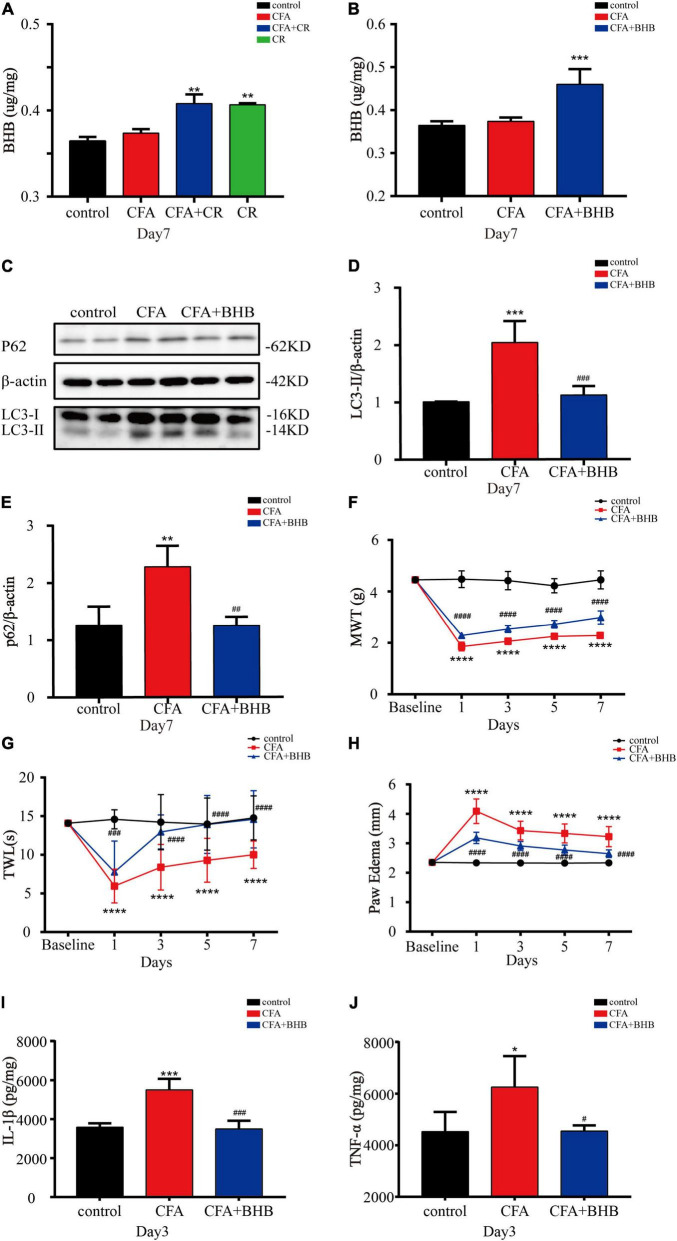
β-Hydroxybutyric acid controls the beneficial effects of CR. **(A)** BHB levels in the spinal cord of mice in the control, CFA, CFA + CR and CR groups. **(B)** BHB levels in the spinal cord of mice in the control, CFA, and CFA + BHB groups after BHB injection. Values are expressed as the mean ± *SD* and were analyzed by one-way analysis of variance, followed by a Turkey’s *post-hoc* test, *n* = 4 per group. ^***^P < 0.001, ^**^P < 0.01, compared to control group; ^###^*P* < 0.001, ^##^*P* < 0.01, ^#^*P* < 0.05, compared to the CFA group. Protein expressions **(C)** of LC3-II **(D)** and p62 **(E)** of mice in the three groups. Values are expressed as the mean ± *SD*, and were analyzed by one-way analysis of variance, followed by a Turkey’s *post-hoc* test, *n* = 4 per group. ^***^P < 0.001, ^**^P < 0.01, compared to control group; ^###^*P* < 0.001, ^##^*P* < 0.01, compared to the CFA group. **(F)** The mechanical withdrawal threshold (MWT) tests, **(G)** the thermal withdrawal latency (TWL) tests and **(H)** the paw edema tests of mice in the control, CFA, and CFA + BHB group. Values are expressed as mean ± *SD* and were analyzed by two-way repeated measures analysis of variance followed by Bonferroni *post-hoc* testing, *n* = 10 per group. ^*⁣*⁣**^P < 0.0001, compared to control group; ^####^*P* < 0.0001, ^###^*P* < 0.001, compared to the CFA group. **(I)** The level of IL-1β and **(J)** TNF-α were measured in the three groups. Values are expressed as mean ± *SD* and were analyzed by one-way analysis of variance, followed by a Turkey’s *post-hoc* test, *n* = 4 per group. ^***^P < 0.001, *P < 0.05, compared to control group; ^###^*P* < 0.001, ^#^*P* < 0.05, compared to the CFA group.

Next, we explored whether BHB controlled the benefits of CR against inflammatory pain. As shown in Figure 5A, BHB levels in the spinal cord were significantly increased in the CFA + CR and CR groups, but CFA administration alone had no impact on the BHB level. These results suggested that CR upregulated BHB levels in the spinal cord. To examine the effect of BHB on inflammatory pain, the mice were injected with BHB. In the CFA + BHB group, BHB levels in the spinal cord increased significantly (Figure 5B). With regard to autophagic flux, we found that LC3-II and p62 levels were decreased in the CFA + BHB group compared with CFA group, while BHB treatment recovered autophagic flux in the ipsilateral spinal cord after CFA administration (Figures 5C–E). As shown above, CR exerted an analgesic effect by restoring autophagic flux in the ipsilateral spinal cord following CFA administration. Therefore, whether BHB also has an analgesic effect on CFA-induced inflammatory pain, its effect on autophagic flux in the spinal cord is similar to that of CR. We found that MWT and TWL were decreased in the CFA + BHB group, and BHB attenuated CFA-induced mechanical and thermal hyperalgesia (Figures 5F,G), indicating that BHB improved CFA-induced inflammatory pain. Additionally, we studied the anti-inflammatory activity of BHB against inflammatory pain. In the CFA + BHB group, CFA-induced paw edema was significantly reduced compared to that in the CFA group (Figure 5H). The levels of the pro-inflammatory factors IL-1β and TNF-α were significantly decreased in mice administered BHB (Figures 5I,J). These results indicate that BHB, as a product of CR, may control the benefits of CR by restoring autophagic flux in the spinal cord.

## Discussion

In order to illustrate the role of CR therapy in CFA-induced inflammatory pain, we must illustrate the following questions. First, what is the role of autophagy in inflammatory pain? Second, does CR improve inflammatory pain by regulating autophagy? Third, how does CR regulate autophagy? MWT and TWL were measured to evaluate the mechanical allodynia and thermal hyperalgesia after CFA injection (Zucoloto et al., 2019). Mice treated with calorie restriction were given 70% of their average daily food intake (Zhang et al., 2020). We studied the autophagy by p62 and LC3-II levels to assess autophagic flux (Yoshii and Mizushima, 2017). Our results show that caloric restriction can improve CFA-induced chronic inflammatory pain by restoring autophagic flux in the spinal cord, and BHB might control the benefits of CR. The findings may show promise for treating chronic inflammatory pain.

It has been demonstrated that CR exerts an analgesic effect in neuropathic pain (Coccurello et al., 2018; Liu et al., 2018; De Angelis et al., 2020). Based on this evidence, we designed an experiment to explore whether CR administration has a protective effect against CFA-induced inflammatory pain. Similar to previous studies (Zucoloto et al., 2019), CFA treatment induced significant mechanical allodynia and thermal hyperalgesia. As demonstrated in chronic constriction injury (CCI) model, our results also show that CR exerts an analgesic effect in CFA-induced inflammatory pain. With regard to the anti-inflammatory activity of CR, CR has been reported to be effective in moderating the expression of some inflammatory markers that are upregulated during aging (Ugochukwu and Figgers, 2007). To study the anti-inflammatory ability of CR in chronic inflammatory pain, paw edema and pro-inflammatory cytokine levels were measured (Nguyen et al., 2020; Xie et al., 2021). CR administration significantly reduced CFA-induced paw edema and pro-inflammatory cytokine levels. To gauge the overall health of mice after CR, our results showed that CR decreased body weight (Figure 2D), which is similar to the results of previous studies (Liu et al., 2018). These findings indicated that CR improved CFA-induced inflammatory pain and showed obvious anti-inflammatory activity.

Previous reports have demonstrated the dysfunction of autophagic flux in SNL and CCI models (Lipinski et al., 2015; Liu et al., 2017). Therefore, we studied the role of autophagic flux in chronic inflammatory pain. Our study found that LC3-II accumulation was accompanied by a significant elevation of p62 in the CFA group. As simultaneous elevation of the autophagy markers LC3-II and P62 (protein substrates degraded by autophagy) indicated impaired autophagic flux. Moreover, CR has been reported to enhance autophagic flux (Zhang et al., 2020). Consistently, we found that additional CR treatment significantly abrogated the CFA-induced upregulation of LC3-II and p62, demonstrating that CR restored CFA-impaired autophagic flux in the ipsilateral spinal cord. To determine whether CR relieved CFA-induced inflammatory pain by improving autophagic flux, CQ, a classic autophagy-lysosome pathway inhibitor, was used in our study. And the analgesic effect of CR diminished when autophagic flux was inhibited. Our results demonstrated that CR improved inflammatory pain by restoring autophagy in the spinal cord.

Previous reports have implicated the protective effects of BHB in various neurodegenerative diseases such as Alzheimer’s disease and Parkinson’s disease (Tieu et al., 2003; Zhang et al., 2013). BHB has been reported to be effective at improving pain hypersensitivity in SCI models (Qian et al., 2017). BHB also stimulates autophagic degradation during glucose deprivation in cultured neurons (Montiel et al., 2020), and has also been found to stimulate autophagic flux in rats (Habieb et al., 2021). Similar to the results of previous studies (Huang et al., 2018), we observed elevated BHB levels after CR therapy. Therefore, we propose that BHB may control the benefits of CR by restoring autophagic flux in CFA-induced inflammatory pain. To verify this hypothesis, we used the DL-BHB. After intraperitoneal injection of BHB for seven consecutive days, the BHB content in the spinal cord increased. BHB administration improved CFA-induced inflammatory pain, reduced the release of pro-inflammatory factors (IL-1β and TNF-α), and restored autophagic flux in the spinal cords of mice with CFA-induced inflammatory pain.

Many studies have been conducted to elucidate the mechanisms underlying inflammatory pain. The present study showed that autophagic flux might influence inflammatory pain. But little is known about how autophagic flux influences inflammatory pain. Pro-inflammatory factors have a vital role in the induction and maintenance in inflammatory pain (Liu et al., 2021). We found elevated TNF-α and IL-1β levels in CFA-induced inflammatory pain, which we hypothesized to be because of impaired autophagic flux in the spinal cord. Our CQ experiment partially verified this hypothesis. Impairing autophagic flux with CQ ameliorated the analgesic effects of CR. Recent studies have observed crosstalk between autophagy and macrophage polarization. Evidence has shown that autophagic flux is an important mechanism for inducing M2 macrophage polarization, and impaired macrophage autophagy promotes pro-inflammatory macrophage polarization in obese mice (Wang et al., 2018). Microglia are the macrophages of the nervous system. Microglial polarization not only participates in central sensitization, but also affects the secretion of proinflammatory cytokines. Therefore, we will focus our studies on how autophagy regulates proinflammatory cytokines and microglial polarization to explore the mechanism by which autophagy regulates chronic inflammatory pain in the future.

**FIGURE 6:**
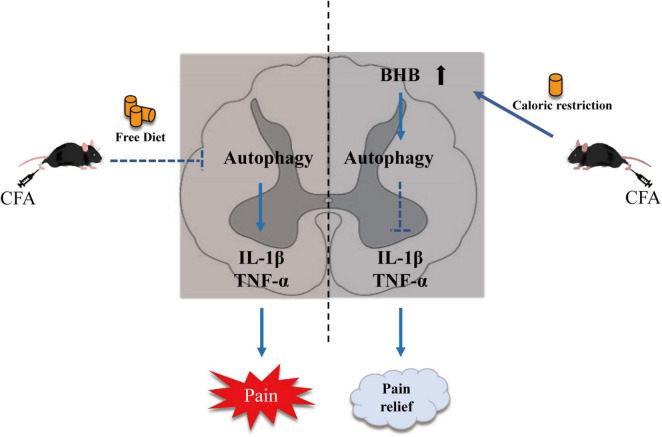
Schematic diagram showing the mechanism by which CR alleviates CFA-induced inflammatory pain. CR elevates β-hydroxybutyric acid expression and restores autophagic flux.

In conclusion, we observed impaired autophagic flux in the spinal cord of mice with CFA-induced inflammatory pain, and subsequently showed that CR improved inflammatory pain by restoring autophagic flux in the spinal cord. Our results also demonstrated that BHB increased in the spinal cord after CR administration and might control the benefits of CR. CR and BHB may provide potential therapeutic interventions for chronic inflammatory pain (Figure 6).

## Data Availability Statement

The original contributions presented in the study are included in the article/Supplementary Material, further inquiries can be directed to the corresponding author/s.

## Ethics Statement

The animal study was reviewed and approved by the Ethical Regulation on the Care and Use of Laboratory Animals of Anhui Medical University.

## Author Contributions

ZY and GX designed the study. CL wrote the manuscript. CL, XZ, LL, YH, QZ, and JZ performed the research and analyzed the data. HW and E-WG revised the manuscript. All authors contributed to the article and approved the submitted version.

## Conflict of Interest

The authors declare that the research was conducted in the absence of any commercial or financial relationships that could be construed as a potential conflict of interest.

## Publisher’s Note

All claims expressed in this article are solely those of the authors and do not necessarily represent those of their affiliated organizations, or those of the publisher, the editors and the reviewers. Any product that may be evaluated in this article, or claim that may be made by its manufacturer, is not guaranteed or endorsed by the publisher.
