# Supplementary material for: Caloric Restriction Alleviates CFA-Induced Inflammatory Pain via Elevating β-Hydroxybutyric Acid Expression and Restoring Autophagic Flux in the Spinal Cord
Source: Front Neurosci. 2022 Apr 28;16:828278. doi: 10.3389/fnins.2022.828278 (PMC9096081; doi:10.3389/fnins.2022.828278)
Supplement: Supplementary file 1 [file Data_Sheet_1.pdf]

Supplementary Material

1 Supplementary Result

Result 1. To test the exact analgesic effect of CR in CFA-induced inflammatory pain, we designed the following experiments. The mice were divided into four groups, control group, CFA group, CFA+CR (seven days) group and CFA+CR (three days) group. The mice in CFA+CR (seven days) group were calorie-restricted for seven days, while the mice in CFA+CR (three days) group were calorie-restricted for three days and were fed ad libitum for the following four days. Then MWT and TWL were measured. There was no significant difference in MWT and TWL between the CFA+CR (seven days) group and the CFA+CR (three days) group on the first and third days after CFA injection. On the fifth and seventh days after CFA injection, the MWT and TWL of the CFA+CR (three days) group were reduced compared with the CFA+CR (seven days) group, suggesting the precise analgesic effect of CR in CFA-induced inflammatory pain (Supplementary material Figure 1A and 1B).

Result 2. In addition, we also used immunofluorescence to detect the expression of LC3 in the ipsilateral spinal cord. Compared with control and CR group, more LC3 positive cells were observed in the L3–5 spinal cord of mice in the CFA group. However, CR decreased the LC3 positive cells after CFA administration (CFA group vs CFA+CR group) (Supplementary material Figure 2A and 2B). These results suggested the blockage of autophagic flux in the ipsilateral spinal cord of mice with CFA-induced inflammatory pain, whereas CR might restore the impaired autophagic flux.

2 Supplementary Figures and Tables

2.1 Supplementary Figures 1

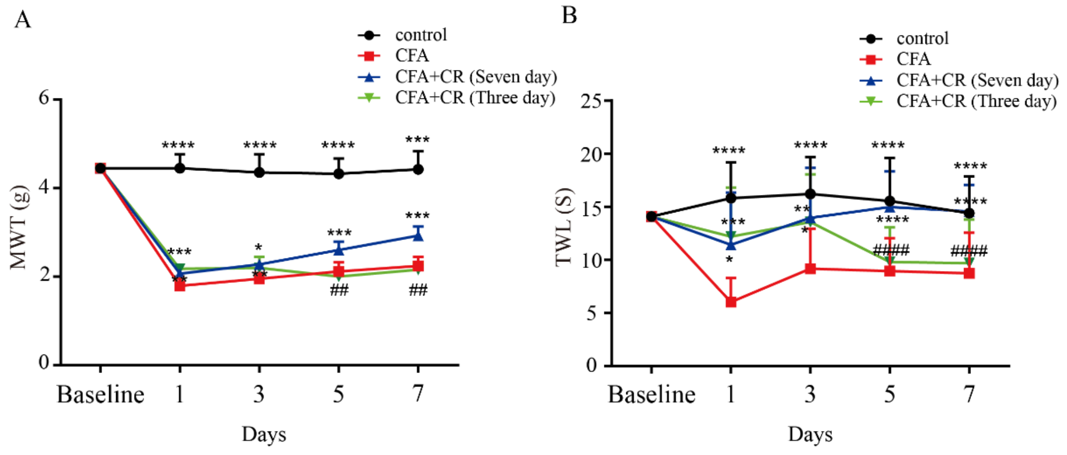

Supplementary material Figure 1 (A) The mechanical withdrawal threshold (MWT)

tests and (B) thermal withdrawal latency (TWL) tests of mice in the control group, CFA group, CFA+CR(seven day) and CFA+CR(three day) group. Values were expressed as the mean ± SD and were analyzed by two-way repeated measures analysis of variance followed by Bonferroni post hoc testing, n = 9 per group. ***P < 0.001, **P < 0.01, *P<0.05, compared to CFA group; $^{###}$P < 0.001, $^{##}$P < 0.01, $^{#}$P < 0.05, compared to the CFA+CR(seven day) group.

2.2 Supplementary Figures 2

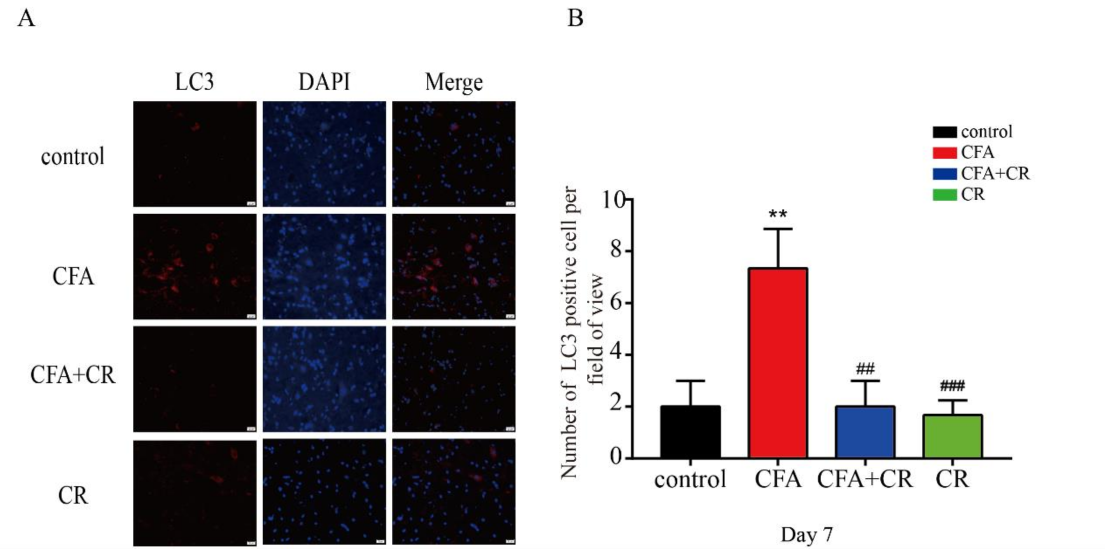

Supplementary material Figure 2 (A) Spinal cords sections immunostained with anti-LC3 antibody, and the nucleus stained with DAPI. Scale bar = 20 μm. (B) Statistical result of the number of LC3 positive cell per field. Values were expressed as mean ± SD and were analyzed by one-way analysis of variance, followed by a Turkey's post hoc test, n = 3 per group. ***P < 0.001, **P < 0.01, *P<0.05, compared to control group; $^{###}$P < 0.001, $^{##}$P < 0.01, $^{#}$P < 0.05, compared to the CFA group.
